# The Application of Template Selectophores for the Preparation of Molecularly Imprinted Polymers

**DOI:** 10.3390/molecules200917601

**Published:** 2015-09-23

**Authors:** Basil Danylec, Lachlan J. Schwarz, Simon J. Harris, Reinhard I. Boysen, Milton T. W. Hearn

**Affiliations:** Centre for Green Chemistry & Australian Centre for Research on Separation Science (ACROSS), School of Chemistry, Monash University, Melbourne, VIC 3800, Australia

**Keywords:** selectophore, template, molecularly imprinted polymer, resveratrol

## Abstract

Molecularly imprinted polymers are versatile materials with wide application scope for the detection, capture and separation of specific compounds present in complex feed stocks. A major challenge associated with their preparation has been the need to sacrifice one mole equivalent of the template molecule to generate the complementary polymer cavities that selectively bind the target molecule. Moreover, template molecules can often be difficult to synthesise, expensive or lack stability. In this study, we describe a new approach, directed at the use of synthetic selectophores, chosen as readily prepared and low cost structural analogues with recognition groups in similar three-dimensional arrangements as found in the target molecule. To validate the approach, a comparative study of selectophores related to the polyphenolic compound (*E*)-resveratrol has been undertaken using traditional and green chemical synthetic approaches. These molecular mimic compounds were employed as polymer templates and also as binding analytes to interrogate the recognition sites associated with the molecularly imprinted polymers. Importantly, the study confirms that the use of selectophores has the potential to confer practical advantages, including access to more efficient methods for selection and preparation of suitable template molecules with a broader range of molecular diversity, as well as delivering imprinted polymers capable of recognizing the target compound and structurally related products.

## 1. Introduction

Historically, waste disposal and recycling of chemicals present at the terminal stages of manufacture within the agricultural and food processing industries have often been perceived as burdens rather than opportunities. In particular, the value-add derived from the recovery and purification of low abundance, but nevertheless very valuable chemicals has rarely been captured as part of integrated unit operations at the level of process efficiencies that are needed to comprehensively address overall supply-chain and productivity considerations. However, within the context of green chemical and engineering technologies, opportunities now exist for industry to deploy new approaches for the separation and recovery of commercially important chemicals from agriculture and food bio-waste sources using non-traditional processes. Some of these considerations have been addressed in our previous investigations, through the development of novel immobilised catalysts and separation materials, which take into account more atom efficient reactions, and through the application of new technologies, which are less resource or energy intensive, for the separation and purification of chemical or biological products.

Within this context, the application of green chemical methodologies for the efficient extraction and separation of naturally occurring bioactive molecules, as an alternative to their chemical synthesis, has considerable potential. For example, in recent studies we have documented a new approach for the separation and purification of bioactive polyphenolic compounds from bio-waste sources, such as grape must and pomace, or peanut husks, using molecularly imprinted polymers (MIPs), with a focus on the phytosterol and polyphenolic compounds, and other valuable plant-derived chemicals [1,2,3,4]. (*E*)-Resveratrol, **1**, is an archetypical example of a phytochemical compound, which arises as an early product from the biosynthesis of phenylalanine. (*E*)-Resveratrol is found in many foodstuffs and is associated with various, important anti-oxidant protective effects [5,6]. Moreover, (*E*)-resveratrol is an intermediate in the biosynthesis of structurally more complicated polyphenols and flavonoids [7].

Based on our previous synthetic studies, analogues of (*E*)-resveratrol were identified as appropriate compounds to validate the concept of selectophores [8] in the generation of molecularly imprinted polymers. Selectophores, as defined by Widstrand *et al.* [8], are template molecules with similar chemical functionalities and geometries—all fitting a similar set of specific properties embodied in a “selectophore” structure, which will bind to the same MIP because of a similar three-dimensional arrangement of interacting functional groups. (*E*)-Resveratrol has a well-defined geometry around the alkene bond, which together with the planar aromatic ring systems, defines the three dimensional orientations and locations occupied by the three hydroxyl groups. These hydroxyl groups function as key sites on this molecule for molecular imprinting, and are recognized by the functional monomers as they self-assemble to form a non-covalent complex as part of the preparation of selective MIPs. The resultant non-covalent complex can then be polymerised with a cross linker to generate the MIP. Upon removal of the template molecule, the imprinted polymer contains complementary cavities that should be able to selectively bind to the original template molecule, or other structurally similar analogues.

From the perspective of green and efficient laboratory practice, the use of a target compound, e.g., (*E*)-resveratrol **1**, as the template to generate the corresponding MIP represents a sacrificial stoichiometric loss, a circumstance which would not be acceptable for large scale MIP-based manufacturing processes, when toxic, high cost or difficult to prepare template molecules are involved. Although the preparation of (*E*)-resveratrol can be achieved, albeit in low yield, by traditional multi-step synthetic methods from available petrochemically-derived precursors, larger template molecules of greater structural complexity often are synthetically more challenging and much more expensive to synthesise. When the issues of time, cost and reagent conservation are taken into account, we were attracted to explore whether template mimics or selectophores [8,9] could be more readily synthesised, and efficiently used, to provide effective substitutes for the target compound of interest. Conceptually, this approach is similar to the use of pharmacophores encountered in rational drug design [10] with the additional advantage that the application of close structural analogues of the parent template for imprinting may overcome the problem of template “bleeding” [11] and the associated uncertainty in trace level analysis after solid-phase extraction with MIPs [12,13,14]. Here, we report the synthesis of the imine **2** and amide **3** selectophores of (*E*)-resveratrol. By utilising green chemical synthetic strategies, these selectophores were more easily and efficiently prepared than (*E*)-resveratrol, with both compounds synthesised in very high yield and purity by “single-pot” reactions. We also report the performances of these new polyphenolic selectophore-imprinted polymers relative to the characteristics found for the corresponding (*E*)-resveratrol-imprinted polymer.

## 2. Results and Discussion

### 2.1. Selectophore Preparations

**Figure 1:**
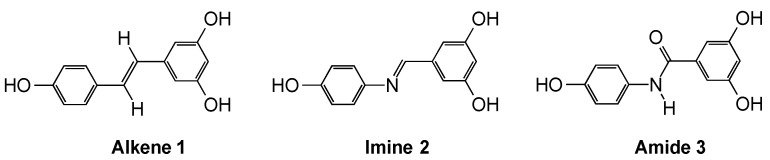
(*E*)-Resveratrol **1** and the polyphenolic selectophores, imine **2** and amide **3**.

(*E*)-Resveratrol **1** is a polyphenolic compound with an alkene constraint. Several analogues were chosen for investigation from the perspective of their ability to replicate this constraint and function as suitable selectophores. The criteria employed were based on their conformational and configurational similarities, as assessed *inter alia* by molecular modelling calculations performed using PM3 geometry optimisation software, and their perceived ease of synthesis. Based on these considerations, the imine, 5-[(4-hydroxy-phenylimino)methyl]-benzene-1,3-diol **2**, and the amide, 3,5-dihydroxy-*N*-(4-hydroxy-phenyl)benzamide **3**, (Figure 1) were selected as potentially useful selectophores. All three compounds have two resorcinol hydroxyls, which are able to occupy the same 3-dimensional spaces, whilst the third phenolic hydroxyl group can exist in a closely aligned overlap position.

**Scheme 1:**
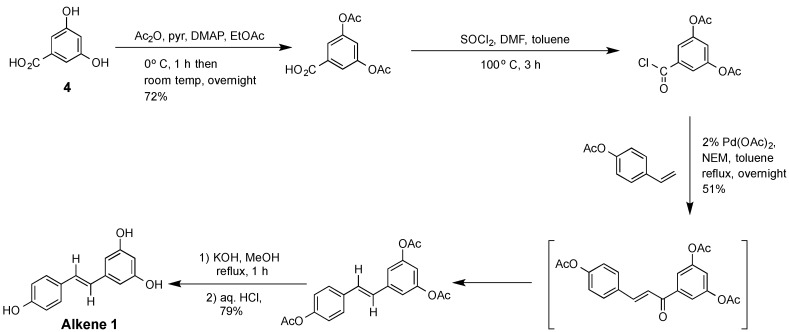
Preparation of the alkene, (*E*)-resveratrol **1**.

In our previous studies, (*E*)-resveratrol was employed as a template molecule for the preparation of several MIPs [2,3,4,15,16]. A variety of traditional methods have been reported [17,18,19,20,21,22] for the synthesis of (*E*)-resveratrol. These methods were not based on the principles of Green Chemistry [23,24], nor did they take into account the requirements of modern industrial practicality. All of these previous synthetic methods invariably employed multistep procedures with significant losses of materials, along with the need for extensive purification processes, repetitive handlings of hazardous reagents, the use of several protection/deprotection steps and significant energy consumption to drive the reactions. Representative of these earlier procedures is the synthesis of (*E*)-resveratrol described by Andrus *et al.* [17] where traditional synthetic protocols are used to transform 3,5-dihydroxybenzoic acid **4** to (*E*)-resveratrol **1** (Scheme 1). As reported in the literature [17,18,19,20,21,22] and replicated in our hands, this multistep procedure for the synthesis of **1** returned a poor atom economy value (*i.e.*, an AEV < 27% if the contributions from all solvents and purification reagents are excluded, or an AEV < 4.3% if the contributions from all chemical components but not the carbon equivalent of the energy used was included), low reaction mass efficiency (2.8% or 0.03%, respectively) and a high E-factor (>26.2 or >3895 respectively), with the desired product obtained in only 29% overall yield. At this laboratory scale typically >5 days were required to complete this synthesis, with handling of a variety of corrosive or hazardous chemicals, as well as the generation of significant waste from the mandatory chromatographic purification steps.

**Scheme 2:**
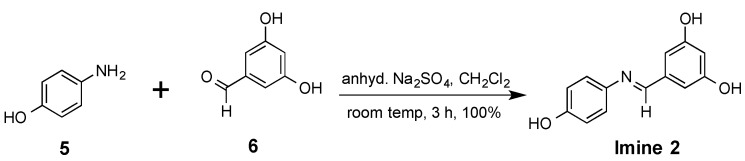
Preparation of the imine 5-[(4-hydroxy-phenylimino)-methyl]-benzene-1,3-diol **2**.

In contrast, the imine selectophore 5-[(4-hydroxy-phenyl-imino)-methyl]-benzene-1,3-diol **2** could be prepared from commercially available 4-aminophenol **5** and 3,5-dihydroxybenzaldehyde **6** as shown in Scheme 2, with an atom economy value of 58%, a reaction mass efficiency of 43.4% and an excellent E-factor of 1.3. By utilising this simple and rapid condensation reaction, nearly quantitative yields of the pure product were rapidly achieved after a simple filtration workup. The stability of the imine **2** was confirmed by ^1^H-NMR spectroscopy studies in *d*_6_-DMSO and CD_3_CN, which showed that this compound remained unchanged in solution at room temperature for at least a 6 day period. This observation was concordant with previous studies [25] with this compound. This imine selectophore was found to also remain intact when used as a template molecule under the conditions employed for MIP preparations, and when used as an analyte to evaluate the binding properties of the various MIPs.

**Scheme 3:**
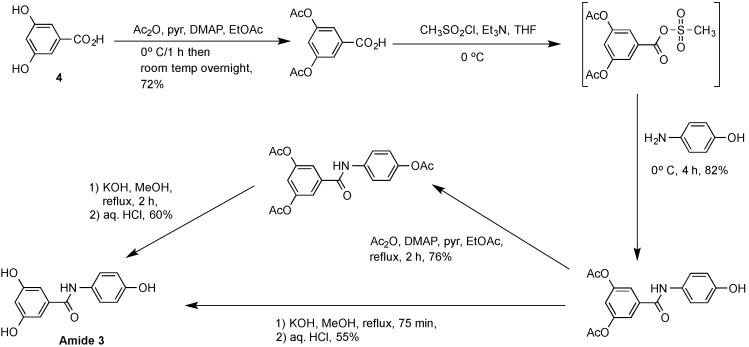
Conventional synthetic method used for the preparation of the amide 3,5-dihydroxy-*N*-(4-hydroxyphenyl)benzamide **3**.

**Scheme 4:**
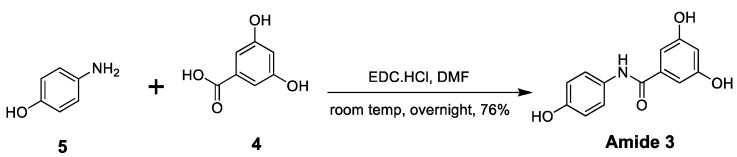
Alternative method for the preparation of the amide 3,5-dihydroxy-*N*-(4-hydroxyphenyl)benzamide **3**.

The second selectophore examined was the amide, 3,5-dihydroxy-*N*-(4-hydroxyphenyl)benzamide **3**. An authentic sample was initially prepared by the multistep procedure (Scheme 3) as adapted from a method briefly outlined as a patent application embodiment by Kim *et al.* [26]. Full experimental details for this multistep synthesis have been included as Electronic Supplementary Information. The conventional multistep preparation of the amide **3** clearly suffered from the same constraints of poor green chemistry metrics with a low atom economy value (*i.e.*, <19.8%) and a high E-factor (in excess of 140) similar to that noted above for the traditional synthesis of (*E*)-resveratrol **1** shown in Scheme 1. Due to our ongoing requirement for larger amounts of this amide **3**, an alternative improved synthetic process was developed, the basis of which is summarized in Scheme 4. Workup of the reaction mixture by removal of the solvent followed by trituration and washing with water rapidly returned the pure amide **3** in 76% yield with an atom economy value of 53.2% and an E-factor of 1.77. The advantage of this carbodiimide-activated coupling procedure is that the same starting reagents were used as for the reported multistep synthesis, yet the amide could be produced in improved overall yield, using a single-pot method, and did not require additional energy, functional group protection and deprotection steps or chromatographic purification.

### 2.2. Molecularly Imprinted Polymers (MIPs)

**Figure 2:**
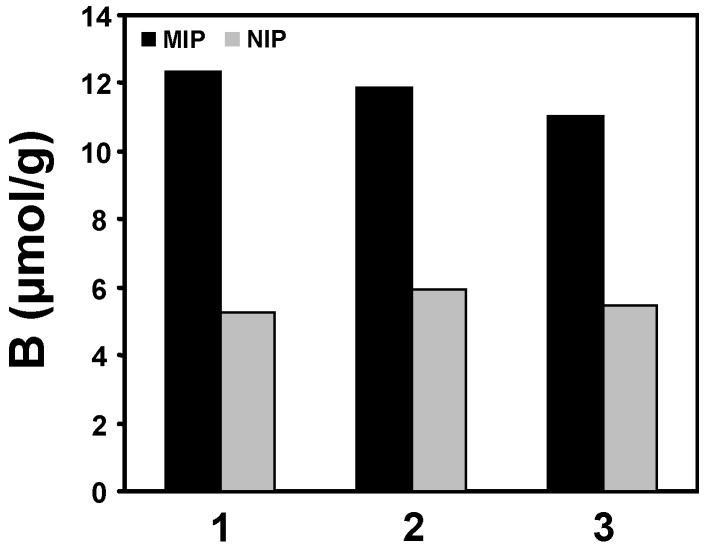
Single analyte binding of (*E*)-resveratrol **1**, or the selectophores, the imine **2**, and the amide **3**, to the MIP_RES_. The standard error of the mean (SEMs) for these binding capacity results was less that ±5% (*n* = 3).

(*E*)-Resveratrol **1** and the two selectophores, **2** and **3**, were used as templates for the preparation of the corresponding MIPs, and as analytes to interrogate the binding characteristics of the newly prepared polymers. The protocols employed for the preparation of such molecularly imprinted polymers (MIPs) have been described in detail elsewhere [2] with the optimal composition of the pre-polymerisation complex obtained using a combination of molecular docking computational and ^1^H-NMR spectroscopy titration procedures. The syntheses of these MIPs, in brief, involved the thermally initiated free radical polymerization of a mixture of the selectophore template, the functional monomer 4-vinylpyridine (4-VP) and the crosslinker in a porogen such as acetonitrile/EtOH (5:1 *v*/*v*). (*E*)-Resveratrol imprinted polymers (MIP_RES_) of similar performance were generated when acetone was used as porogen, with all other variables kept constant. The resultant solid materials were ground, appropriately sized particles recovered by sieving and then washed with methanolic acetic acid to desorb the template. Washings were monitored using RP-HPLC with UV-Vis detection, and repeated until no traces of the template were detected. Non-imprinted polymers (NIPs) were prepared in an analogous manner in the absence of template molecule. Static binding studies were performed using the MIP_RES_ and the corresponding NIP with individual analytes. In these studies, the polymers were incubated with a freshly prepared solution of the analyte, the supernatant removed, the concentration of the free analyte in the supernatant determined by RP-HPLC measurements, and the amount of analyte bound (B) by the polymer determined. MIP blanks were examined without any added analytes present and demonstrated no template bleed. The results are summarized in Figure 2.

As evident from these results, the MIP_RES_ displayed similar binding capacities and comparable selectivities (B_MIP_ − B_NIP_) for the alkene, the amide and the imine molecules. The similarity of these binding parameters suggests that the MIP_RES_ displays a generic three-point binding cross-reactivity towards each of these polyphenolic compounds. This result is concordant with all three molecules having similar conformations and configurations in solution and within the micro-environment of the MIP cavity. Moreover, it is interesting to note that although the two selectophores, **2** and **3**, are likely to possess similar electronic characteristics as the parent (*E*)-resveratrol, due to the nature of the differences between the three bridging linkages (alkene, imine and amide), inductive variations appear to be moderated by both the distance from the aromatic hydroxyl groups and the electronic nature of the aromatic π clouds. Consequently, although these selectophores appear to bind in a similar manner with MIP_RES_ at the same cavity recognition sites as (*E*)-resveratrol, differences in affinity occur, as manifested by the subtle variation in their binding capacities.

**Figure 3:**
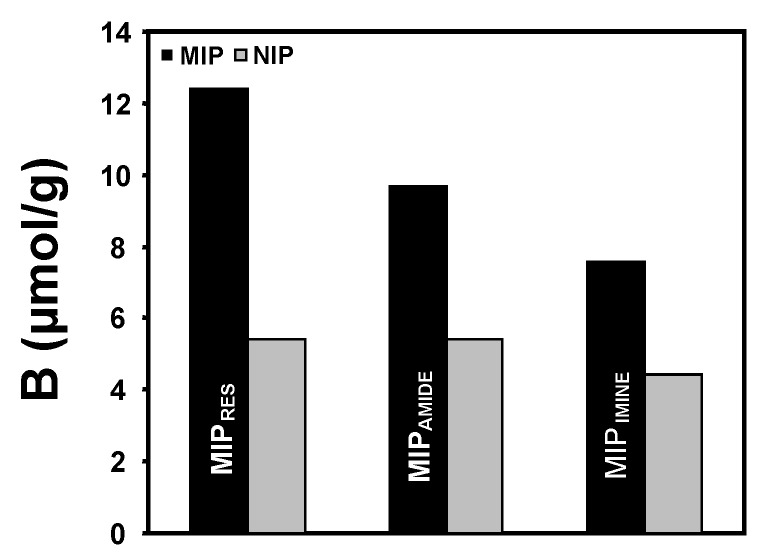
Recognition of (*E*)-resveratrol by MIP_RES_, MIP_AMIDE_ and MIP_IMINE_. The standard error of the mean (SEMs) for these binding capacity results was less that ±5% (*n* = 2).

Based on these results, the suitability of the amide and imine selectophores as (*E*)-resveratrol template mimics was then assessed using similar static binding assays. Thus, both of these compounds were employed as templates for the preparation of new MIPs (*i.e.*, MIP_AMIDE_ and MIP_IMINE_ respectively) using similar protocols to that employed for the preparation of MIP_RES_. The ability of these MIPs to recognise (*E*)-resveratrol is shown in Figure 3.

As evident from Figure 3, the selectophore-imprinted MIP_AMIDE_ and MIP_IMINE_ both displayed enhanced affinity for (*E*)-resveratrol compared to the corresponding NIPs, but with the structural rigidity of this compound due to the alkene constraint reduced capacities compared to MIP_RES_. Despite the fact that all three polyphenolic compounds may have similar conformations in solution, these findings indicate that the stereo-topological spaces occupied by the pyridyl nitrogens of the 4-VP monomer units within the polymers, despite acting as complementary functional binding sites for hydrogen bond formation during the imprinting process and for the subsequent molecular recognition phenomenon with the polyphenols, must differ slightly for all three MIPs. These differences results in slightly lower binding capacities and affinities for both MIP_AMIDE_ and MIP_IMINE_ with (*E*)-resveratrol, a finding concordant with the conformation of (*E*)-resveratrol being more constrained due to the presence of its alkene moiety and less able to be fully accommodated in the selectophore-generated binding cavities

## 3. Experimental Section

### 3.1. Reagents and Equipment

Reagents and other chemicals were purchased from Sigma-Aldrich (Castle Hill, NSW, Australia) or Merck (Kilsyth, VIC, Australia). AR solvents were used as obtained from the manufacturer except for dimethylformamide (DMF), which was dried over 4 Å molecular sieves, toluene was dried over sodium wire, and pyridine and triethylamine were dried over KOH pellets. Milli-Q distilled water was used for aqueous solutions. Solvent mixtures are expressed as volume/volumes. Solvent extracts were dried over anhydrous sodium sulfate, filtered and then rotary evaporated to dryness at low pressures (≥30 mbar) at 30–35 °C. Reaction mixtures were sonicated in a 37 kHz/150 W Elmasonic S100 ultrasonic bath. Polymers were ground using a Retsch PM 200 planetary ball mill (Haan, Germany) and sized through sieves on a Retsch AS 200 sieve shaker. Analytical thin layer chromatography (TLC) was conducted on silica gel using Merck^®^ 1.05554.001 plates (Merck, Darmstadt, Germany). The components were visualised by: (i) fluorescence at 254 nm; and (ii) ethanolic phosphomolybdic acid solution dip and char. Silica gel column chromatography was conducted using Merck^®^ 1.09385.1000. Nuclear magnetic resonance spectra were recorded on a Bruker DPX-300 spectrometer (^1^H at 300 MHz and ^13^C JMOD at 75 MHz) (Bruker, Billerica, MA, USA). Deuterated solvents were used as indicated and the residual solvent peaks used for internal reference. Coupling constant *J* values are reported in Hz (hertz). Low resolution electrospray ionisation mass spectra (ESI) were recorded on a Micromass platform II API QMS electrospray mass spectrometer in both positive (ESI^+^) and negative (ESI^−^) polarity modes. High-resolution electrospray mass spectra (HRMS) were recorded on a Bruker BioApex 47e Fourier Transform mass spectrometer. Melting points were determined using a Büchi melting point B-545 apparatus with a digital thermometer by open glass capillary method and are uncorrected.

### 3.2. Syntheses of Polyphenolic Selectophores

#### 3.2.1. 3,5-Diacetoxybenzoic Acid

A suspension of 3,5-dihydroxybenzoic acid (15.40 g, 0.100 mol) in ethyl acetate (220 mL) was cooled in an ice-bath. Acetic anhydride (24.52 mL, 0.2421 mol), pyridine (16.16 mL, 0.1998 mol) and 4-(dimethylamino)pyridine (100 mg, 0.8186 mmol) were added and the reaction stirred at 0 °C for 60 min and then at room temperature overnight. Formic acid (5.12 mL, 0.1357 mmol) was added and the reaction poured onto ice (*ca.* 500 g). The volume was increased by the addition of further ethyl acetate (300 mL) and the organic phase separated and washed with water (2 × 200 mL), sat. aq. NaHCO_3_ (100 mL), further water (2 × 200 mL), and then dried and evaporated to a white solid. Recrystallization of the product from 5:1 (*v*/*v*) EtOAc/hexane (120 mL) gave 2 crops of 3,5-diacetoxybenzoic acid (combined weight 17.07 g, 72%) as a white powder. R*_f_* 0.20 (1:1 EtOAc/hexane), 0.39 (3:1 EtOAc/hexane); mp 161–162 °C (lit. [27] mp: 157–159 °C); δ_H_ (CDCl_3_) 2.29 (s, 6H, 2 × OAc), 7.18 (pseudo t, 1H, *J* = 2.1 Hz, 4-H) and 7.70 (pseudo d, 2H, *J* = 2.1 Hz, 2-H, 6-H); δ_C_ (CD_3_OD) 18.43, 118.94, 119.02, 131.70, 150.19, 165.46 and 168.17; *m*/*z* (ESI) 261 ([M + Na]^+^, 100%).

#### 3.2.2. (*E*)-3,4′,5-Triacetoxystilbene

A suspension of 3,5-diacetoxybenzoic acid (8.022 g, 33.71 mmol) in a mixture of toluene (130 mL), DMF (500 μL) and thionyl chloride (16.00 mL, 220.6 mmol) was heated at 100 °C for three hours under an argon gas atmosphere. The solvents were removed by vacuum distillation and the residue re-suspended in toluene (85 mL) and sonicated under vacuum to remove dissolved gases. 4-Acetoxystyrene (5.74 mL, 37.5 mmol), *N*-ethylmorpholine (4.31 mL, 33.9 mmol) and palladium diacetate (35 mg, 0.16 mmol, 0.46 mol %) were added and the reaction heated to reflux for 2 h. Further palladium diacetate (116 mg, 0.52 mmol, 1.54 mol %) was added and the reaction left to reflux overnight. On return to room temperature, ethyl acetate (500 mL) was added, the solution washed with 0.1 M aq. HCl (2 × 300 mL) and water (300 mL) and then dried and evaporated to return a brown solid. Purification with column chromatography (isocratically eluted with 2:1 Et_2_O/hexane) gave 7.888 g of a white solid, shown by ^1^H-NMR spectroscopy to be predominantly the desired adduct. Further chromatography (gradient eluted starting with 4:1 hexane/EtOAc and finishing with 2:1 hexane/EtOAc) returned pure (*E*)-3,4′,5-triacetoxystilbene (6.071 g, 51%) as a white solid. R*_f_* 0.29 (2:1 hexane/EtOAc); mp 112.5–113.0 °C (lit. [17] mp 116 °C); δ_H_ (CDCl_3_) 2.27 (s, 9H, 3 × OAc), 6.80 (pseudo t, 1H, *J* = 2.1 Hz, 4′-H), 6.93 (d, 1H, *J* = 16.3 Hz, H*_trans_*), 7.03 (d, 1H, *J* = 16.3 Hz, H*_trans_*), 7.04–7.09 (m, 4H, 3-H, 5-H, 2′-H, 6′-H) and 7.44–7.47 (m, 2H, 2-H, 6-H); δ_C_ (CDCl_3_) 20.07, 113.39, 115.88, 120.88, 126.19, 126.64, 128.64, 133.45, 138.53, 149.46, 150.34, 167.91 and 168.30; *m*/*z* (ESI) 377 ([M + Na]^+^, 100%), 378 (21%).

#### 3.2.3. (*E*)-Resveratrol, **1**

The synthesis was conducted under an argon gas atmosphere. Potassium hydroxide (22 mg, 0.3922 mmol) was dissolved in methanol (3.0 mL) and this solution added to a suspension of (*E*)-3,4′,5-triacetoxystilbene (113 mg, 0.319 mmol) in methanol (10 mL). The solid immediately dissolved and the clear solution was gently heated to reflux for 60 min, with an associated progressive darkening in colouration. The volume was then reduced to half by rotary evaporation and the remaining solution acidified (pH 2) with 1 M aq. HCl. Ethyl acetate (150 mL) was added and the reaction washed with saturated brine (3 × 20 mL), dried and evaporated to return a dark red solid. Purification with column chromatography (isocratically eluted with 100% EtOAc) gave (*E*)-resveratrol (58 mg, 79%) as a pale beige coloured solid. R*_f_* 0.65 (EtOAc); mp 261.0–263.0 °C (lit. [18] mp 255–260 °C); δ_H_ (CD_3_OD) 6.13 (pseudo t, 1H, *J* = 2.2 Hz, 4-H), 6.41–6.42 (m, 2H, 2-H, 6-H), 6.71–6.79 (m, 3H, H*_trans_*, 3′-H, 5′-H), 6.93 (d, 1H, *J* = 16.3 Hz, H*_trans_*) and 7.29–7.36 (m, 2H, *J_ortho_* = 8.6 Hz, 2′-H, 6′-H); δ_C_ (CD_3_OD) 100.30, 103.47, 114.12, 124.64, 126.43, 127.06, 128.07, 138.97, 155.89 and 157.20; *m*/*z* (ESI) 229 ([M + H]^+^, 100%), 230 (23%).

#### 3.2.4. 5-[(4-Hydroxy-phenylimino)-methyl]-benzene-1,3-diol, **2**

A mixture of 3,5-dihydroxybenzaldehyde (300 mg, 2.174 mmol), 4-aminophenol (237 mg, 2.174 mmol), anhydrous sodium sulphate (309 mg, 2.176 mmol) and dichloromethane (15 mL) was vigorously stirred at room temperature for 3 h. Further anhydrous sodium sulphate (309 mg, 2.176 mmol) was added and the stirring continued for an additional one hour. Dichloromethane was removed by rotary evaporation and the white residue re-suspended in boiling ethanol (10 mL) and filtered whilst hot. The solid was washed in the funnel with further hot ethanol (10 mL). The clear filtrates were combined and rotary evaporated to dryness to give 5-[(4-hydroxy-phenylimino)-methyl]-benzene-1,3-diol (501 mg, 100%) as a pale pink solid. R*_f_* 0.48 (2:1 EtOAc/hexane); darkens with heating with mp >340 °C; δ_H_ (*d*_6_-DMSO) 6.36 (pseudo t, 1H, *J* 2.2, 4-H), 6.80–6.85 (m, 4H, 2-H, 6-H, 3′-H, 5′-H), 7.16–7.22 (m, 2H, *J_ortho_* = 8.8 Hz, 2′-H, 6′-H), 8.43 (s, 1H, imine-H), 9.47 (bs, 3H, 3 × phenolic-OH); δ_C_ (*d*_6_-DMSO) 106.27, 107.41, 116.70, 123.42, 139.35, 143.61, 157.16, 158.38 and 159.62; *m*/*z* (ESI) 230 ([M + H]^+^, 100%), 231 (13%); *m*/*z* (HRESI) 252.0633 ([M + Na]^+^, C_13_H_11_NO_3_Na^+^ requires 252.0637).

#### 3.2.5. 3,5-Dihydroxy-*N*-(4-hydroxyphenyl)benzamide, **3**

The reaction was conducted under an argon gas atmosphere. 3,5-Dihydroxybenzoic acid (2.310 g, 15.0 mmol) and 4-aminophenol (1.962 g, 18.0 mmol) were dissolved in DMF (90.0 mL). *N*-Ethyl-*N*′-(3-dimethylaminopropyl)carbodiimide hydrochloride (3.450 g, 17.997 mmol) was added and the reaction stirred at room temperature overnight. The solvent was removed by rotary evaporation under high vacuum and the oily residue co-evaporated with water until a solid formed. This solid was triturated with cold 0.01 M aq. HCl (50 mL), filtered, and the pale purple coloured product washed with cold water (4 × 20 mL). The solid was dried over desiccant under vacuum to give pure 3,5-dihydroxy-*N*-(4-hydroxyphenyl)benzamide (2.791 g, 76%); R*_f_* 0.39 (4:1 EtOAc/hexane); mp 266.0–266.5 °C; δ_H_ (CD_3_OD) 6.47 (pseudo t, 1H, *J_meta_* 2.2, 4-H), 6.77–6.82 (m, 4H, 3′-H, 5′-H, 2-H, 6-H), 7.43–7.46 (m, 2H, *J_ortho_* = 8.9 Hz, 2′-H, 6′-H); δ_H_ (*d*_6_-DMSO) 6.42 (pseudo t, 1H, *J_meta_* = 2.2 Hz, 4-H), 6.72–6.78 (m, 4H, 3′-H, 5′-H, 2-H, 6-H), 7.51–7.56 (m, 2H, *J_ortho_* = 8.9 Hz, 2′-H, 6′-H), 9.20 (s, 1H), 9.51 (s, 2H) and 9.83 (s, 1H); δ_C_ (CD_3_OD) 104.41, 104.67, 113.92, 122.13, 129.10, 136.08, 153.26, 157.41 and 166.70; *m*/*z* (ESI) 244 ([M − H]^−^, 100%), 489 ([2M − H]^−^, 29%); *m*/*z* (HRESI) 246.0764 ([M + H]^+^, C_13_H_12_N_1_O_4_^+^ requires 246.0766).

### 3.3. Preparation of Molecularly Imprinted Polymers

Molecularly imprinted polymers (MIPs) were prepared by procedures described previously using an optimised composition for the pre-polymerisation complex, calculated from the average energy of formation for the template, monomer 4-vinylpyridine (4-VP) clusters and monomer-template clusters, using semi-empirical equilibrium geometry level theory and PM3 force field calculations [28,29]. The reliability of these determinations was then confirmed using ^1^H-NMR spectroscopy titration experiments by monitoring the ^1^H-NMR spectrum of the template molecule (0.1 mM), dissolved in trideuteroacetonitrile (CD_3_CN) and titrated with increasing molar equivalents of 4-VP. Spectra were recorded after each addition. This process was continued until the aromatic –OH signal was no longer detectable due to peak broadening. Thus, in brief, the selectophore template **2** (1 mmol) was dissolved in the porogen (5:1 acetonitrile/EtOH, 6 mL) in a disposable glass test tube. For the amide **3**, acetone (6 mL) was the preferred porogen due to the lower solubility of this selectophore in 5:1 acetonitrile/EtOH. The functional monomer 4-VP (322 μL, 3 mmol) was added and the mixture sonicated for 10 min. Ethylene glycol dimethacrylate (2.314 mL, 15 mmol) was then added as cross-linker, and 2,2′-azobisisobutyronitrile (51 mg, 0.31 mmol) added as radical initiator. This pre-polymerisation mixture was sparged with nitrogen gas for 5 min before being heated in a thermostatic water bath at 50 °C for 24 h. The solid polymers were removed from the reaction tubes, crushed with a mortar and pestle and finely ground using a Retsch 200 ball mill. The powder was sieved and the 63–100 μm size particles recovered. These particles were repeatedly suspended in ethanol or acetone and the supernatant decanted to remove fines. The bound template was then removed from the polymer by repeated washings with methanolic 10% acetic acid until the washings were free of template (monitored by UV-Vis spectroscopy at 321, 270 and 280 nm for compounds **1**, **2** and **3**, respectively). Non-imprinted control polymers (NIPs) were prepared using the same procedure in the absence of a template molecule.

### 3.4. Evaluation of Molecularly Imprinted Polymer

Static binding experiments were conducted with both the MIPs and NIPs in parallel under identical conditions. The analyte solutions (1.5 mL, 0.5 mM in acetonitrile) were added to the polymers (30 mg) in Eppendorf tubes. This suspension was mixed at 40 rpm for 18 h and the polymer pelleted by centrifugation for 15 min at 13,000 rpm. A 200 μL aliquot of the supernatant was removed and analysed by RP-HPLC with UV detection at 321, 270 and 280 nm for compounds **1**, **2** and **3**, respectively. The concentration of the non-bound analyte was determined by comparison with a linear 5-point calibration curve. The amount of analyte bound (B) was then determined as the difference between this non-bound value and the initial value, and expressed in units of μmol analyte bound/g polymer. MIP blanks, using 1.5 mL of acetonitrile without any added analyte present to assess the extent of template bleeding [11], were also employed as controls.

## 4. Conclusions

This study has documented that structural analogues with spatially defined functionalities similar to a parent compound can be used as selectophores or template mimics for the preparation of molecularly imprinted polymers (MIPs). Using the polyphenol (*E*)-resveratrol as exemplar, such polymeric materials were found to display comparable, but not identical, binding behaviour towards this representative polyphenolic compound. These findings indicate that by mimicking the structural and stereo-electronic properties of a parent compound, appropriate selectophores can be designed and employed as suitable templates, enabling the convenient preparation of generic MIPs which are capable of binding not only to the parent molecule, but also structurally similar compounds. This approach thus provides an avenue to readily explore the binding characteristics of new MIPs, particularly when the availability, structural complexity or instability of the parent molecule limits its use as a template. One such example where the utilisation of these concepts would be beneficial is with the structurally more complex, but highly valued, flavonoid and phytosterol compounds present in low abundance in plant waste materials. Since in many cases the possibility exists for such selectophores to be more easily and conveniently prepared than the target compounds using single-pot methods, or with other green chemical synthetic procedures, increased application of selectophores in the development of MIP technologies in molecular sensing devices, compound purification and waste stream processing can thus be anticipated.

## Supplementary Materials

Supplementary materials can be accessed at: http://www.mdpi.com/1420-3049/20/09/17601/s1.
