# Supplementary material for: The Application of Template Selectophores for the Preparation of Molecularly Imprinted Polymers
Source: Molecules. 2015 Sep 23;20(9):17601–13. doi: 10.3390/molecules200917601 (PMC6332243; doi:10.3390/molecules200917601)
Supplement: Supplementary file 1 [file molecules-20-17601-s001.pdf]

Supplementary Information

S1. Introduction

Experimental procedures for the conventional synthesis of the amide selectophore 3,5-dihydroxy-*N*-(4-hydroxyphenyl)benzamide **3** described below have been adapted from information provided for a multistep procedure that was originally outlined as a partial description within a brief embodiment as part of a patent application by Kim *et al.* [26] (Scheme 3). Our results are summarized below with the reference numbering in this Electronic Supplementary Information kept the same as found in the text of the main paper.

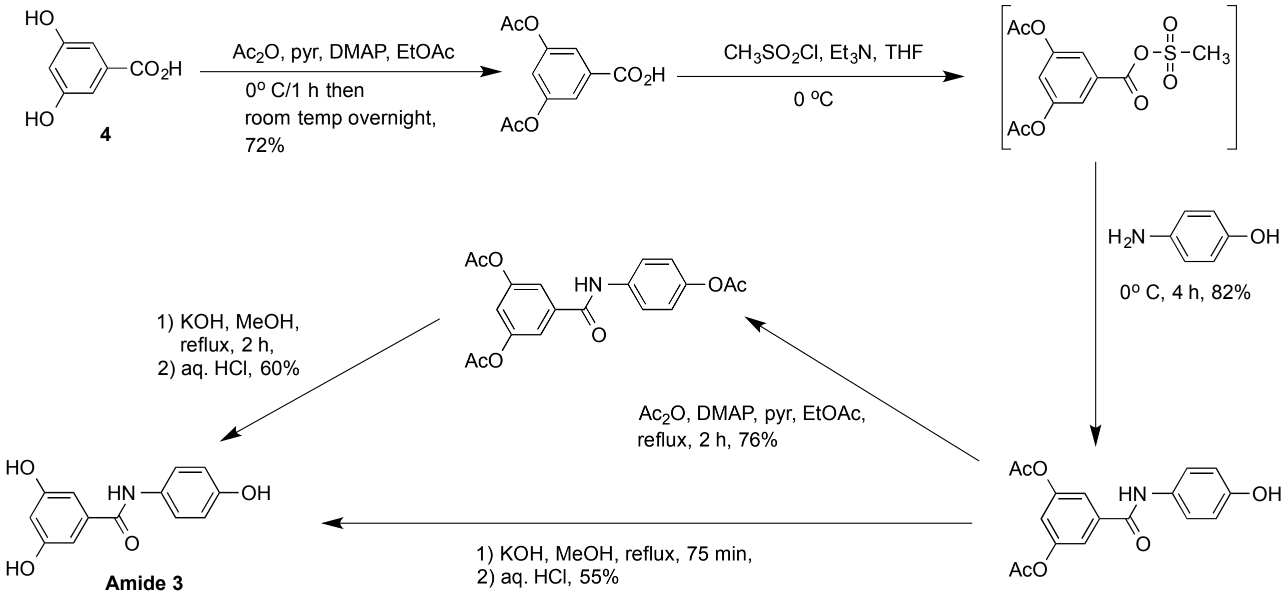

Scheme 3. Reagents, conditions and product yields obtained for the preparation of the amide selectophore **3** and its precursor intermediates.

S2. Experimental Section

S2.1. Preparation of 3,5-Diacetoxybenzoic Acid

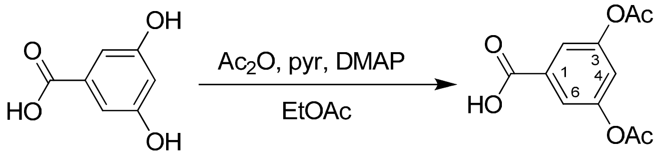

Acetic anhydride (24.52 mL, 0.2421 mol), 4-(dimethylamino)pyridine (100 mg, 0.81855 mmol) and pyridine (16.16 mL, 0.1998 mol) were added to a suspension of 3,5-dihydroxybenzoic acid (15.40 g, 0.100 mol) in ethyl acetate (220 mL), cooled in an ice-bath. The reaction mixture was stirred at 0 °C for 60 min and then overnight at room temperature. Formic acid (5.12 mL, 0.1357 mol) was added and the reaction mixture poured onto ice (*ca.* 500 g). Ethyl acetate (300 mL) was added and the organic phase recovered, washed with water (2 × 200 mL), sat. aq. $NaHCO_3$ (100 mL), further water (2 × 200 mL), dried (anhyd. Na_2SO_4), filtered and rotary evaporated to return a white solid. Recrystallization of the product from 5:1 EtOAc/hexane (120 mL) gave 2 crops of 3,5-diacetoxybenzoic acid (combined weight 17.073 g, 72% yield) as a white powder. R_f 0.20 (1:1 EtOAc/hexane), 0.39 (3:1 EtOAc/hexane); mp

161–162 °C (lit. [27] mp: 157–159 °C); ^{1}H-NMR ($CDCl_3$): δ 2.29 (s, 6H, 2 × OAc), 7.18 (pseudo t, 1H, *J* 2.1 Hz, 4-H), 7.70 (pseudo d, 2H, *J* = 2.1 Hz, 2-H, 6-H); ^{13}C JMOD NMR (CD_3OD): δ 18.43 (2 × CH_3), 118.94 (4), 119.02 (2,6), 131.70 (1), 150.19 (5), 165.46 (COOH), 168.17 (2 × $OCOCH_3$); LRESI positive ion mass spectrum; *m/z* 261 ($[M + Na]^+$, 100%).

S2.2. Alternative Method for the Preparation of 3,5-Diacetoxybenzoic Acid

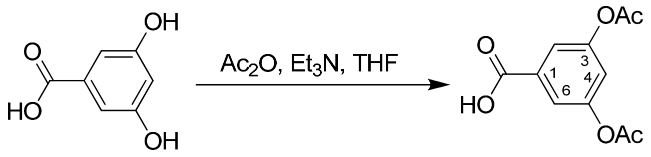

3,5-Dihydroxybenzoic acid (7.700 g, 0.050 mmol) was dissolved in THF (120 mL). Triethylamine (19.0 mL, 0.1358 mol) was added and a white solid immediately precipitated out off solution. The reaction was stirred for 10 min and then acetic anhydride (12.5 mL, 0.12 mol) was added drop wise over 10 min. The reaction was heated to reflux for 4 h, cooled to room temperature, and more acetic anhydride (5.0 mL, 0.048 mol) added. The yellow solution was left to stand overnight. The THF was removed by rotary evaporation and replaced with dichloromethane (500 mL), and the solution successively washed with water (3 × 200 mL), 1M aq. HCl (3 × 300 mL), and water (3 × 200 mL). The organic phase was dried (anhyd. Na_2SO_4), filtered and rotary evaporated to give a white solid (8.539 g). Trituration with hexane (2 × 20 mL) gave the product after drying as a white solid (7.034 g). This product was recrystallized from 1:1 EtOAc/hexane (60 mL) to return 3,5-diacetoxybenzoic acid as two crops of identical white powder (with a combined mass of 4.060 g) in 34% yield. The characterization data for this product was identical to that for the earlier preparation.

S2.3. Preparation of 5-(4-Hydroxyphenylcarbamoyl)-1,3-phenylene Diacetate

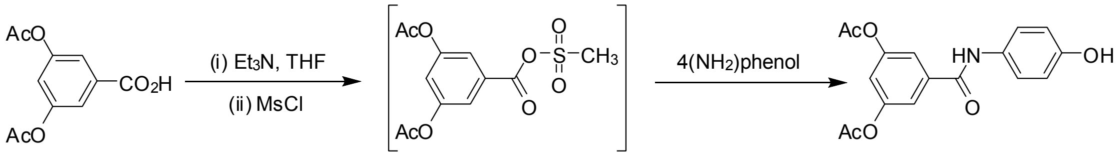

The reaction was conducted under a positive pressure of argon gas and in an ice-bath at 0 °C. Triethylamine (1.344 mL, 8.403 mmol, dried over KOH pellets) was added to a solution of 3,5-di-acetoxybenzoic acid (2.000 g, 8.403 mmol) in anhydrous THF (35.0 mL). Methanesulphonyl chloride (672 μL, 8.403 mmol) was slowly syringed into the solution over 5 minutes and the reaction mixture stirred for a further 25 minutes. 4-Aminophenol (1.008 g, 8.403 mmol) was then added and the reaction mixture well stirred at 0 °C for a further 4 h prior to storage overnight at −20 °C. The reaction mixture was then acidified (pH 2) with 1 M HCl, the solvents removed by rotary evaporation, and the residual yellow gum azeotropically dried by repeated additions of ethanol and rotary evaporation. The residual gum was dissolved in EtOAc (150 mL), washed with water (4 × 50 mL), dried (anhyd. Na_2SO_4), filtered and rotary evaporated to give a white solid (2.815 grams). Attempted purification by column chromatography (0.040–0.063 mm SiO_2, isocratically eluted with 2:1 EtOAc/hexane) gave the crude 5-(4-hydroxyphenylcarbamoyl)-1,3-phenylene diacetate as a white solid (2.260 g) in 82% yield. R_f 0.56

(2:1 EtOAc/hexane). ^{1}H-NMR (CD_3OD) showed this preparation to be ≤75% pure. In order to conserve reagents, this material was used in the next steps of the synthesis.

S2.4. Preparation of 5-(4-Acetoxyphenylcarbamoyl)-1,3-phenylene Diacetate

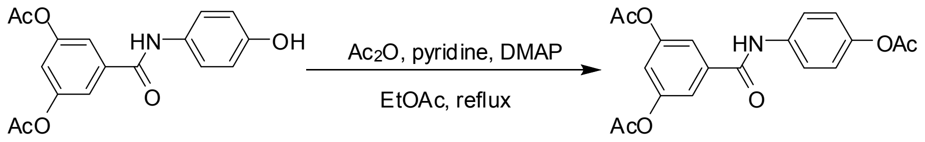

The "crude" 5-(4-hydroxyphenylcarbamoyl)-1,3-phenylene diacetate (602 mg, 1.830 mmol, ≤75% pure) was suspended in ethyl acetate (20 mL). 4-(Dimethylamino)pyridine (20 mg, 0.164 mmol), pyridine (3.00 mL, 37.092 mmol) and acetic anhydride (3.00 mL, 31.737 mmol) were added. Within 5 min all solids had dissolved. The clear solution was then heated to reflux for 2 hours and left to stand at room temperature overnight. Further ethyl acetate (100 mL) was added and the organic solvent fraction of the reaction mixture was washed with 0.1 M HCl (2 × 50 mL) and water (2 × 50 mL), dried (anhyd. Na_2SO_4), filtered and rotary evaporated to return a white solid (640 mg). Due to poor solubility, the solid was re-dissolved in ethyl acetate (100 mL), 6.0 g of silica added, and the solvent rotary evaporated off. The remaining powder was loaded onto a silica column as a dry plug and the product chromatographed (0.040–0.063 mm SiO_2, with gradient elution beginning with 2:1 EtOAc/hexane and finishing with 5:1 EtOAc/hexane) to give 516 mg (76% yield) of 5-(4-acetoxyphenylcarbamoyl)-1,3-phenylene diacetate, obtained as a white powder. R_f 0.63 (2:1 EtOAc/hexane). ^{1}H-NMR (d_6-DMSO) showed this to be *ca.* 80% pure and it was used as is for the ongoing hydrolysis reaction.

S2.5. Preparation of 3,5-Dihydroxy-N-(4-hydroxyphenyl)benzamide by Hydrolysis of the Diacetate

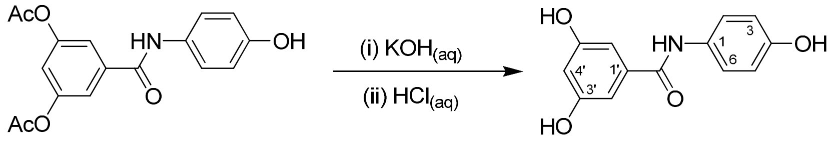

The reaction was conducted under a positive pressure of argon gas. A solution of potassium hydroxide (842 mg, 15.006 mmol) in water (30.0 mL) was added to the "crude" 5-(4-hydroxyphenylcarbamoyl)-1,3-phenylene diacetate (750 mg, 2.280 mmol, ≤75% pure). The solid dissolved within 5 min at room temperature. The pale yellow solution was then heated to reflux for 75 min. On return to room temperature, 1 M aq. HCl was added until a precipitate formed (pH 3). This solid was filtered off and repeatedly washed in a Buckner funnel with water (8 × 20 mL). Thorough drying of the product under vacuum returned 3,5-dihydroxy-*N*-(4-hydroxyphenyl)benzamide (310 mg, 55% yield) as fluffy small white needles. R_f 0.39 (4:1 EtOAc/hexane); mp: 266.0–266.5 °C; ^{1}H-NMR (CD_3OD): δ 6.47 (pseudo t, 1H, J_{meta} = 2.2 Hz, 4-H), 6.77-6.82 (m, 4H, 3′-H, 5′-H, 2-H, 6-H), 7.43–7.46 (m, 2H, J_{ortho} = 8.9 Hz, 2′-H, 6′-H); ^{1}H-NMR (d_6-DMSO): δ 6.42 (pseudo t, 1H, J_{meta} = 2.2 Hz, H-4), 6.72–6.78 (m, 4H, H-3′, H-5′, H-2, H-6), 7.51–7.56 (m, 2H, J_{ortho} = 8.9 Hz, 2′-H, 6′-H), 9.20 (s, 1H), 9.51 (s, 2H), 9.83 (s, 1H); ^{13}C-JMOD-NMR (CD_3OD): δ 104.41 (C4), 104.67 (2,6), 113.92 (3′,5′), 122.13 (2′,6′), 129.10 (1′), 136.08 (1), 153.26(4′), 157.41 (3,5), 166.70 (C=O); LRESI negative ion mass spectrum; *m*/*z* 244

([M − H]$^-$, 100%), 489 ([2M − H]$^-$, 29%), HRESI positive ion mass spectrum; *m*/*z* for $C_{13}H_{11}NO_4$, calculated [M + H]$^+$ 246.0766, measured 246.0764.

S2.6. Preparation of 3,5-Dihydroxy-N-(4-hydroxyphenyl)benzamide by Hydrolysis of the Triacetate

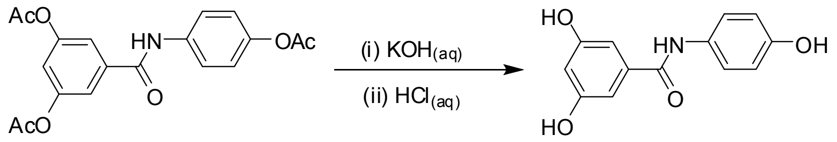

The reaction was conducted under a positive pressure of argon gas. A solution of potassium hydroxide (1.684 g, 30.012 mmol) in water (60.0 mL) was added to the "crude" 5-(4-acetoxyphenylcarbamoyl)-1,3-phenylene triacetate (1.456 g, 4.4255 mmol, ≤75% pure). The reaction was heated to reflux for 2 h. On return to room temperature, 1 M HCl was slowly added until a precipitate formed (pH 3). This solid was filtered off and repeatedly washed in a Buckner funnel with water (8 × 30 mL). The white solid was then dried under vacuum to give 3,5-dihydroxy-*N*-(4-hydroxyphenyl)benzamide (653 mg, 60% yield) as fluffy small white needles. Melting point and spectroscopic data were identical to the previously prepared material.

S3. References

Reference numbering in this Electronic Supplementary Information has been kept the same as found in the text of the main paper.

26. Kim, S.J.; Baek, H.S.; Rho, H.S.; Kim, D.H.; Chang, I.S.; Lee, O.S.; Shin, H.J. Preparation of Hydroxybenzamides as Antiaging and Antiwrinkle Cosmetic Ingredients. WO2007021067A1, 22 Feburary 2007.
27. Turner, S.R.; Voit, B.I.; Mourey, T.H. All-aromatic hyperbranched polyesters with phenol and acetate end groups: Synthesis and characterization. *Macromolecules* **1993**, *26*, 4617–4623.
